# Commentary: Electromagnetic navigation with nonintubated thoracoscopic wedge resection: Summating novelties to reveal truths

**DOI:** 10.1016/j.xjtc.2021.10.053

**Published:** 2021-10-29

**Authors:** Matthew Egyud, Bryan M. Burt

**Affiliations:** Michael E. DeBakey Department of Surgery, Baylor College of Medicine, Houston, Tex

**Figure:**
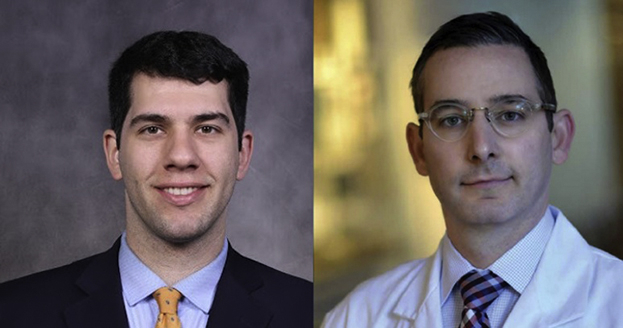
Matthew Egyud, MD, and Bryan M. Burt, MD

**Central Message**

Single-stage localization and appropriate resection procedures for certain suspicious lung nodules remain advantageous.

See Article page 517.

In their manuscript, Hsu and colleagues^1^ retrospectively evaluate patients who underwent electromagnetic navigation–guided intraoperative nodule localization and uniportal video-assisted thoracoscopic surgery (VATS) pulmonary resection. Some patients underwent nonintubated anesthesia, and some patients underwent endotracheal tube anesthesia. It appears that the primary objective of this study was to study the impact of nonintubated anesthesia on a workflow that includes intraoperative electromagnetic navigation localization through the study of perioperative outcomes. Compared with 25 patients in the intubated group, the 21 patients in the nonintubated group spent an expectedly shorter amount of time in the operating room (20 minutes on average). Further demonstrated was equivalency of postoperative outcomes between the intubated and nonintubated group, including postoperative morbidity and length of chest tube drainage. As expected, patients in the nonintubated group developed a greater respiratory acidosis during surgery than patients who were intubated; however, these physiologic findings did not translate into meaningful differences clinical course.

Seemingly a secondary objective of this study was the comparative efficacy of electromagnetic navigational bronchoscopy (ENB) in intubated versus nonintubated patients. Using metrics of fluorescent green signals on the pleural surface overlying the injected lesion and the avoidance of finger palpation, success of localization was observed to be similar in each group. The demonstration of equivalency of navigation in intubated versus nonintubated patients, in so much as could be demonstrated cohorts of 21 and 25 patients in size, is a potential contribution of this study. In other words, the method of anesthesia did not affect the efficacy of ENB-guided intraoperative localization. Following localization, lung nodules were removed by wedge resection in 96% of the patients in this study, and these nodules were found to be malignant in 90% of cases. Unknown is how measures of interest would stand up in a workflow that is appropriately enriched for anatomic resection.

Showcased in this manuscript is the novelty in combining ENB intraoperative localization with uniportal VATS pulmonary resection and with nonintubated anesthesia. Nonintubated uniportal VATS wedge resection was not shown to be superior to intubated uniportal VATS wedge resection with regard to intraoperative or postoperative outcomes, and feasibility for ENM in nonintubated uniportal lobectomy is suggested. The advantages of a one-stage procedure for localization and resection of a suspicious lung nodule, after thoughtful patient selection and careful operative planning that includes appropriate extent of resection, remain advantageous.
